# Supplementary material for: Single laser pulse generates dual photoacoustic signals for differential contrast photoacoustic imaging
Source: Sci Rep. 2017 Apr 4;7:626. doi: 10.1038/s41598-017-00725-4 (PMC5429673; doi:10.1038/s41598-017-00725-4)
Supplement: Supplementary file 1 — Supplementary materials [file 41598_2017_725_MOESM1_ESM.pdf]

Supplementary information

Single laser pulse generates dual photoacoustic signals for differential contrast photoacoustic imaging

Fei Gao[1,2,†], Xiaohua Feng[1,†], Ruochong Zhang[1], Siyu Liu[1], Ran Ding[1], Rahul Kishor[1], Yuanjin Zheng[1,*]

[1]*School of Electrical and Electronic Engineering, Nanyang Technological University, Singapore*

[2]*School of Information Science and Technology, ShanghaiTech University, Shanghai, China*

[†]Authors contributed equally to this work

[*]Corresponding to: yjzheng@ntu.edu.sg

1. Derivation of Equation (2)

Eq. (2) represents the long laser pulse induced PA signal. Compared with the conventional short laser-pulse induced PA signal p_1, the incremental signal strength originates from two parts: absolute temperature (AT) increase induced PA signal enhancement Δp_AT, and accumulated heat difference (HD) Δp_HD between object and surround medium:

$$p_2 = p_1 + \Delta p\_\mathrm{AT} + \Delta p\_\mathrm{HD} \quad \textbf{(S1)}$$

The absolute temperature term Δp_AT could be expressed as:

$$\Delta p\_\mathrm{AT} = \overbrace{b\eta_{th}\mu_a\tau_{th}^2\phi\left[1-\left(1+\frac{\Delta t}{\tau_{th}}\right)e^{-\frac{\Delta t}{\tau_{th}}}\right]}^{Gruneisen\ saturation\ term}\eta_{th}\mu_a\phi\delta t \quad \textbf{(S2)}$$

Which refers to the PA signal increase due to absolute temperature related Gruneisen parameter increase, whose Gruneisen saturation term could be derived from below note 2.

The heat difference term Δp_HD could be expressed as below depending on laser pulse width:

$$\Delta p\_\mathrm{HD} = \begin{cases} \Gamma_0\eta_{th}\mu_a \overbrace{\phi\Delta t}^{Fluence}\text{, short laser pulse, thermal confinement satisfied} \\ \Gamma_0\eta_{th}\mu_a \overbrace{\phi\Delta t}^{Fluence}\ \overbrace{e^{-\frac{\Delta t}{\tau_{th}}}}^{Thermal\ diffusion}\text{, long laser pulse, thermal confinement unsatisfied} \end{cases} \quad \textbf{(S3)}$$

where the top expression is the conventional short-pulse PA generation with thermal confinement satisfied, which is linearly proportional to the laser fluence term. The bottom expression refers to the long pulse case, the heat difference will also be influenced by concurrent heat diffusion during the long laser illumination.

By incorporating Eq. (S1)-(S3), the final expression of long laser pulse induced PA signal could be expressed as:

$$p_2=\left\{\Gamma_0+\overbrace{b\eta_{th}\mu_a\tau_{th}^2\phi\left[1-\left(1+\frac{\Delta t}{\tau_{th}}\right)e^{-\frac{\Delta t}{\tau_{th}}}\right]}^{\text{Gruneisen saturation term}}\right\}\eta_{th}\mu_a\phi\delta t+\Gamma_0\eta_{th}\mu_a\overbrace{\phi\Delta t}^{\text{Fluence}}\overbrace{e^{-\frac{\Delta t}{\tau_{th}}}}^{\text{Thermal diffusion}} \qquad \textbf{(S4)}$$

2. Derivation of the Gruneisen saturation term

The Gruneisen saturation term is derived by integrating the heat deposition and diffusion over laser pulse width:

$$
\begin{aligned}
&\text{Gruneisen saturation term} \\
&= b\eta_{th}\mu_a\phi\int_0^{\Delta t} te^{-\frac{t}{\tau_{th}}}dt \\
&= b\eta_{th}\mu_a\phi\left(-\tau_{th}\int_0^{\Delta t} tde^{-\frac{t}{\tau_{th}}}\right) \\
&= b\eta_{th}\mu_a\phi\left[-\tau_{th}\left(te^{-\frac{t}{\tau_{th}}}-\int_0^{\Delta t} e^{-\frac{t}{\tau_{th}}}dt\right)\right] \\
&= b\eta_{th}\mu_a\phi\left[-\tau_{th}\left(te^{-\frac{t}{\tau_{th}}}+\tau_{th}e^{-\frac{t}{\tau_{th}}}\right)\right]\Bigg|_0^{\Delta t} \\
&= b\eta_{th}\mu_a\phi\left[-\tau_{th}^2\left(\frac{t}{\tau_{th}}e^{-\frac{t}{\tau_{th}}}+e^{-\frac{t}{\tau_{th}}}\right)\right]\Bigg|_0^{\Delta t} \\
&= b\eta_{th}\mu_a\phi\left[-\tau_{th}^2\left(1+\frac{t}{\tau_{th}}\right)e^{-\frac{t}{\tau_{th}}}\right]\Bigg|_0^{\Delta t} \\
&= b\eta_{th}\mu_a\phi\left[-\tau_{th}^2\left(1+\frac{\Delta t}{\tau_{th}}\right)e^{-\frac{\Delta t}{\tau_{th}}}+\tau_{th}^2\right] \\
&= b\eta_{th}\mu_a\phi\tau_{th}^2\left[1-\left(1+\frac{\Delta t}{\tau_{th}}\right)e^{-\frac{\Delta t}{\tau_{th}}}\right]
\end{aligned}
\tag{S5}
$$

3. Derivative of Equation (3)

The derivative of Eq. (3) is derived to find the maximum amplitude of the nonlinear PA signal:

$$
\begin{aligned}
\frac{\partial p_2}{\partial \Delta t} &= \frac{\partial\left\{p_1 + B\tau_{th}^2\left[1-\left(1+\frac{\Delta t}{\tau_{th}}\right)e^{-\frac{\Delta t}{\tau_{th}}}\right] + C\Delta t e^{-\frac{\Delta t}{\tau_{th}}}\right\}}{\partial \Delta t} \\
&= \frac{\partial\left\{p_1 + B\tau_{th}^2 - B\tau_{th}^2\left(1+\frac{\Delta t}{\tau_{th}}\right)e^{-\frac{\Delta t}{\tau_{th}}} + C\Delta t e^{-\frac{\Delta t}{\tau_{th}}}\right\}}{\partial \Delta t} \\
&= \frac{\partial\left\{p_1 + B\tau_{th}^2 - B\tau_{th}^2 e^{-\frac{\Delta t}{\tau_{th}}} - B\tau_{th}\Delta t e^{-\frac{\Delta t}{\tau_{th}}} + C\Delta t e^{-\frac{\Delta t}{\tau_{th}}}\right\}}{\partial \Delta t} \\
&= \frac{\partial\left(p_1 + B\tau_{th}^2\right)}{\partial \Delta t} + \frac{\partial\left(-B\tau_{th}^2 e^{-\frac{\Delta t}{\tau_{th}}}\right)}{\partial \Delta t} + \frac{\partial\left[\left(C - B\tau_{th}\right)\Delta t e^{-\frac{\Delta t}{\tau_{th}}}\right]}{\partial \Delta t} \\
&= 0 + \left(-B\tau_{th}^2\right)\left(-\frac{1}{\tau_{th}}\right)e^{-\frac{\Delta t}{\tau_{th}}} + \left(C - B\tau_{th}\right)\left[e^{-\frac{\Delta t}{\tau_{th}}} + \left(-\frac{1}{\tau_{th}}\right)e^{-\frac{\Delta t}{\tau_{th}}}\Delta t\right] \\
&= B\tau_{th}e^{-\frac{\Delta t}{\tau_{th}}} + Ce^{-\frac{\Delta t}{\tau_{th}}} - \frac{C}{\tau_{th}}e^{-\frac{\Delta t}{\tau_{th}}}\Delta t - B\tau_{th}e^{-\frac{\Delta t}{\tau_{th}}} + Be^{-\frac{\Delta t}{\tau_{th}}}\Delta t \\
&= \left[C + \left(B - \frac{C}{\tau_{th}}\right)\Delta t\right]e^{-\frac{\Delta t}{\tau_{th}}}
\end{aligned}
\qquad \textbf{(S6)}
$$

4. Characterization of IR-820

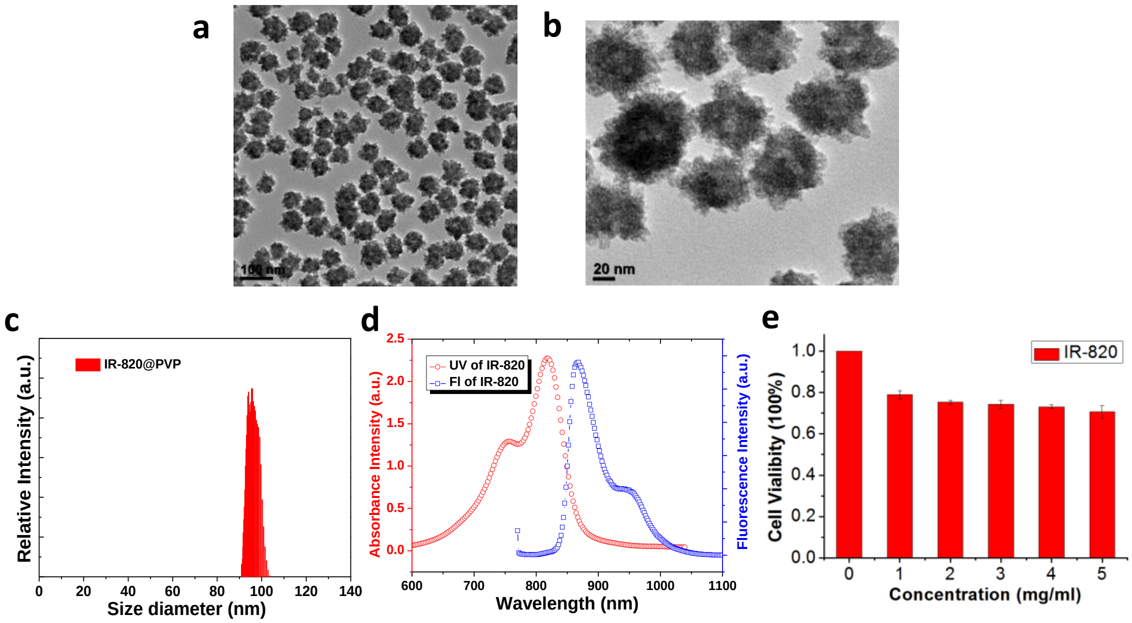

Figure S1 (a-b) TEM images of dispersed IR-820 in aqueous solution. (c) The nanoparticle size distribution of dispersed IR-820. (d) UV absorbance and fluorescence of IR-820 with a concentration of 1×10-2 mM. (e) Quantitative MTT analysis showing the cell viability when incubating NIH3T3 cells with IR-820.
